# Increased Brain Tissue Oxygen Monitoring Threshold to Improve Hospital Course in Traumatic Brain Injury Patients

**DOI:** 10.7759/cureus.7115

**Published:** 2020-02-27

**Authors:** Tye Patchana, James Wiginton, James Brazdzionis, Hammad Ghanchi, Bailey Zampella, Harjyot Toor, Ryan Dorkoski, Anjali Mannickarottu, Margaret Wacker, Raed Sweiss, Dan E Miulli

**Affiliations:** 1 Neurosurgery, Riverside University Health System Medical Center, Moreno Valley, USA; 2 Environmental and Plant Science, Ohio University, Athens, USA; 3 Neurosurgery, Arrowhead Regional Medical Center, Colton, USA

**Keywords:** cerebral tissue oxygenation, traumatic brain injury, pbto2, brain tissue oxygen monitoring, tbi

## Abstract

Introduction

This article is a retrospective analysis of the neurosurgical census at our institution to determine an optimal threshold for brain tissue oxygenation (PbtO2). The use of brain tissue oxygen monitoring has been in place for approximately three decades but data suggesting optimal thresholds to improve outcomes have been lacking. Though there are multiple modalities to monitor cerebral oxygenation, the monitoring of brain tissue oxygen tension has been deemed the gold standard. Still, it is not clear exactly how reductions in PbtO2 should be treated or what appropriate thresholds to treat might be. The aim of our study was to determine if our threshold of 28 mmHg for a good functional outcome could be correlated to the Glasgow Coma Scale (GCS) and Glasgow Outcome Scale (GOS).

Methods

A retrospective analysis of the Arrowhead Regional Medical Center (ARMC) Neurosurgery Census was performed. Patients from 2017-2019 who had placement of Licox® cerebral oxygen monitoring sensors (Integra® Lifesciences, Plainsboro Township, New Jersey) were included in the analysis. Fifteen patients were consecutively identified, all of which presented with traumatic brain injury (TBI). Data on age, gender, days in the intensive care unit (ICU), days before discharge or end of medical care, admission GCS, hospital length of stay, GOS, maximum and minimum PbtO2 values for five days following insertion, minimum and maximum intracranial pressures (ICPs), and brain temperature were included for analysis. Patient data were separated into two groups; those with consistently higher PbtO2 scores (≥ 28 mmHg; n = 7) and those with inconsistent/lower PbtO2 scores (< 28 mmHg; n = 8). Standard student t-tests were used to find potential statistical differences between the groups (α = 0.05).

Results

There were seven patients in the consistently high PbtO2 category (≥ 28 mmHg) and eight patients in the inconsistent/low PbtO2 category (<28 mmHg). The average maximum and minimum PbtO2 for the group displaying worse outcomes (as defined by GCS/GOS) was 23.0 mmHg and 14 mmHg, respectively. Those with consistent Day 2 PbtO2 scores of ≥ 28 mmHg had significantly higher GCS scores at discharge/end of medical care (p < 0.05). Average GCS for the patient group with >28 mmHg PbtO2 averaged over Days 2-5 group was 11.4 (n=7). Average GCS for the <28 group was 7.0 (n=8). The GCS for the >28 group was 63% higher than found in the <28 group (p = 0.03). GOS scores were significantly higher in those with consistently higher PbtO2 (≥ 28) than those with lower PbtO2 scores (< 28). The averages were 3.5 in the higher PbtO2 group as compared to 2 in the lower PbtO2 group.

Conclusion

Along with ICP monitors and monitoring in the assessment of CPP, brain tissue oxygenation allows yet another metric by which to optimize treatment in TBI patients. At our institution, a PbtO2 level of ≥ 28 mmHg is targeted in order to facilitate a good functional outcome in TBI patients. Keeping patients at this level improves GCS and GOS at discharge/end of medical treatment.

## Introduction

Given the observation that episodes of hypoxemia and ischemia following severe head injury increased mortality, the advent of brain tissue oxygenation monitoring in the 1990s had become an important tool in the intensive care unit (ICU) to avoid these insults [1-2]. Along with ICP monitors and monitoring in the assessment of cerebral perfusion pressure (CPP), brain tissue oxygenation allows yet another metric by which to optimize treatment in TBI patients. Previous methods of assessment of cerebral hypoxia relied on jugular bulb oximetry. As opposed to placement within the jugular bulb during jugular bulb oximetry or transcranial cerebral oximetry utilizing near-infrared light, brain tissue oxygen monitoring is predicated on the insertion of a polarographic microcatheter into the frontal white matter. Although all of these modalities have helped to decrease the mortality associated with TBI, evidence is lacking to inform a clear threshold below which aggressive treatment to increase PbtO2 should be initiated.

The Licox® brain tissue oxygenation system (Integra® Lifesciences, Plainsboro Township, New Jersey) was introduced into clinical practice in 1993, and initially saw most of its use in the setting of traumatic brain injury (TBI) and subarachnoid hemorrhage (SAH) [3]. The system is able to measure both oxygen and temperature in the intracranial cavity, with the intention of providing additional metrics to inform clinical decision-making and augmentation of treatment. Typically, the more severely injured TBI patients receive brain tissue oxygen monitoring. These patients usually already have ICP monitoring placed, and the placement of a PbtO2 probe is an additional metric by which to obtain information guiding treatment. Indeed, the indications for brain tissue oxygenation monitoring are similar to those for ICP monitoring. Elevated ICP in the setting of low PbtO2 may portend that further intervention or augmentation of current treatment is required for the patient to maximize functional outcomes (as indicated by Glasgow Coma Score (GCS) and Glasgow Outcome Scale (GOS)). This may include changes to the ventilation settings, changes in blood pressure parameters that affect cerebral perfusion pressure, changes in sedation, or the need for surgical intervention.

Though the use of brain tissue oxygen monitoring has been in place for close to three decades with improvement in outcomes, data suggesting optimal thresholds to change outcomes have been lacking [4-5]. Though there are multiple modalities to monitor cerebral oxygenation, including jugular venous oxygen saturation and transcranial cerebral oximetry, the monitoring of brain tissue oxygen tension has been deemed the gold standard [6].

Previously, correlations between reduced brain tissue oxygenation and poor outcome in TBI patients have been observed [4]. Studies have set PbtO2 < 15 mmHg as the threshold for the prognostication of increased morbidity and mortality [7]. Indeed, the Brain Trauma Foundation guidelines also recommend supporting PbtO2 above 15 mmHg [1]. The currently accepted goal of therapy at most institutions aim to keep PbtO2 > 15-20 mmHg [8]. At our institution, we consider a partial oxygen pressure above 20 mmHg to be indicative of functional outcome and aim for readings above 28 mmHg for a good outcome [9].

## Materials and methods

A retrospective analysis of the Arrowhead Regional Medical Center (ARMC) Neurosurgery Census was performed. Patients from 2017-2019 who had placement of Licox cerebral oxygen monitoring sensors were included in this analysis. Fifteen patients were consecutively identified, all of which presented with traumatic brain injury (TBI). Data on age, gender, days in ICU, days before discharge/end of medical care, admission GCS, GCS at discharge/end of medical care, GOS, maximum and minimum PbtO2 values for five days following insertion, minimum and maximum ICPs, and brain temperature were included. Patients were separated by those with consistently high post Day 2 PbtO2 (> 28 mmHg) measurements from those with inconsistent and/or lower post Day 2 PbtO2 scores (< 28 mmHg). Day 2 PbtO2 values were chosen in order to allow the Licox brain tissue monitoring device a period of calibration.

We set the minimum requirement to be binned into the consistently high PbtO2 group as having at least ≥ 28 mmHg PbtO2 maximum for Days 3, 4, and 5 (must meet that requirement for all three days). This was the defined threshold below which to treat at our institution to attain a good functional outcome. Seven patients were identified with consistently higher PbtO2 scores (≥ 28 mmHg), and eight patients were identified with inconsistent/lower PbtO2 scores (< 28 mmHg). Standard t-tests were used to find potential statistical differences between the consistently high and inconsistently high PbtO2 groups. All statistical analysis, plot, and graph construction was done using Microsoft Excel (2016) with the Analysis ToolPak (Microsoft Corporation, Redmond, Washington).

## Results

**Figure 1:**
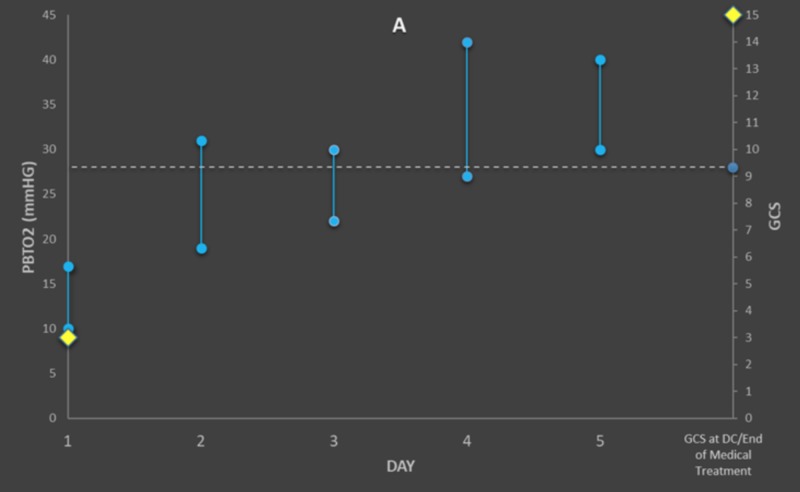
Graph for Patient A, demonstrating maximum and minimum PbtO2 levels above the pre-defined 28 mmHg threshold (dotted line) GCS during the first day of monitoring and at the hospital discharge or the end of ICU-level care are demonstrated by yellow diamonds. Glasgow Coma Scale (GCS), brain tissue oxygenation (PBtO2)

The most common cause of TBI in our patient analysis occurred via motor vehicle accidents. Out of 15 patients, there were three deaths (20%). Our analysis included more men (n=11, 73%) than women (n=4, 28%), and most patients were within their first three decades of life. All patients were intubated, either in the pre-hospital setting or shortly after arrival, and were admitted to the ICU. Licox monitors were placed following initial stabilization typically within the first 24-48 hours of admission. PbtO2 levels were recorded hourly by nursing staff. Daily maximum and minimum values were obtained for the first five days of cerebral tissue oxygenation monitoring (Figure 1). This was based on the Integra LifeScience technical literature directions for use of the complete brain probe kit [10].

Patient data (15 patients, labeled A-O) were separated by those with consistently high post Day 2 PbtO2 (≥ 28 mmHg) measurements from those with inconsistent and/or lower post Day 2 PbtO2 scores (< 28 mmHg) (Table 1). The minimum requirement to be binned into the consistently high PbtO2 group was having a maximum value of at least 28 mmHg for Days 3, 4, and 5. There were seven patients in the consistently high PbtO2 category (top of Table 1) and eight patients in the inconsistent/low PbtO2 category (bottom of Table 1).

**Table 1:** Displays our patient population separated into two groups The consistently high PbtO2 group (above) is represented by seven patients (A-M) who had maximum PbtO2 > 28 mmHg over Days 2-5. The inconsistently high/low PbtO2 group (below) is represented by eight patients (B-O), who did not have maximum PbtO2 > 28 mmHg over Days 2.5. Brain tissue oxygenation (PBtO2)

		Day 1		Day 2		Day 3		Day 4		Day 5	
Group	Patient	Min	Max	Min	Max	Min	Max	Min	Max	Min	Max
Consistently High PbtO2 (> 28 mmHg)	A	10	17	19	31	22	30	27	42	30	40
	C	29	34	31	38	28	41	31	42	31	42
	F	11	32	36	39	18	40	21	31	22	30
	H	13	17	19	32	19	30	27	39	28	39
	J	30	33	30	55	29	38	29	36	37	50
	K	13	29	22	31	22	31	22	31	22	31
	M	14	60	29	38	20	44	24	42	30	39
Inconsistent/low PbtO2 (< 28 mmHg)	B	6	9	6	9	6	9	6	9	27	30
	D	24	54	24	54	22	27	22	27	21	25
	E	3	10	8	17	9	16	7	17	7	18
	G	10	24	13	25	15	27	10	21	11	20
	I	5	13	8	16	8	15	8	15	8	25
	L	19	19	18	51	11	28	6	33	11	19
	N	8	22	9	18	9	18	20	32	21	32
	O	4	24	25	30	28	35	29	32	NA	NA

**Figure 2:**
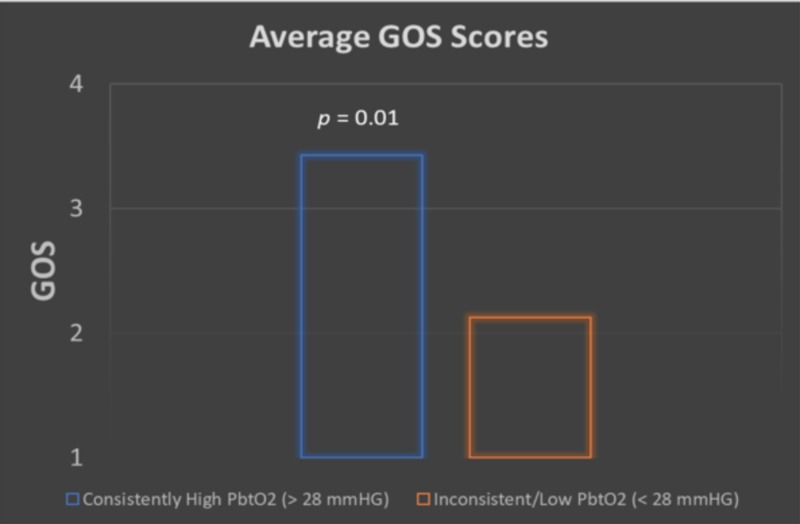
Bar graph displaying average GOS for the consistently high PbtO2 group (blue) and the inconsistent/low PbtO2 group (orange) Average GOS for the consistently high group was 3.4 (n = 7); average GCS for the inconsistent/low group was 2.1 (n=8) Glasgow Outcome Score (GOS), brain tissue oxygenation (PBtO2)

The graphical display of the data tabulated for each patient is shown in Figure 2. All maximum and minimum PbtO2 data over the first five days, as well as initial and discharge GCS are displayed (Appendix).

**Figure 3:**
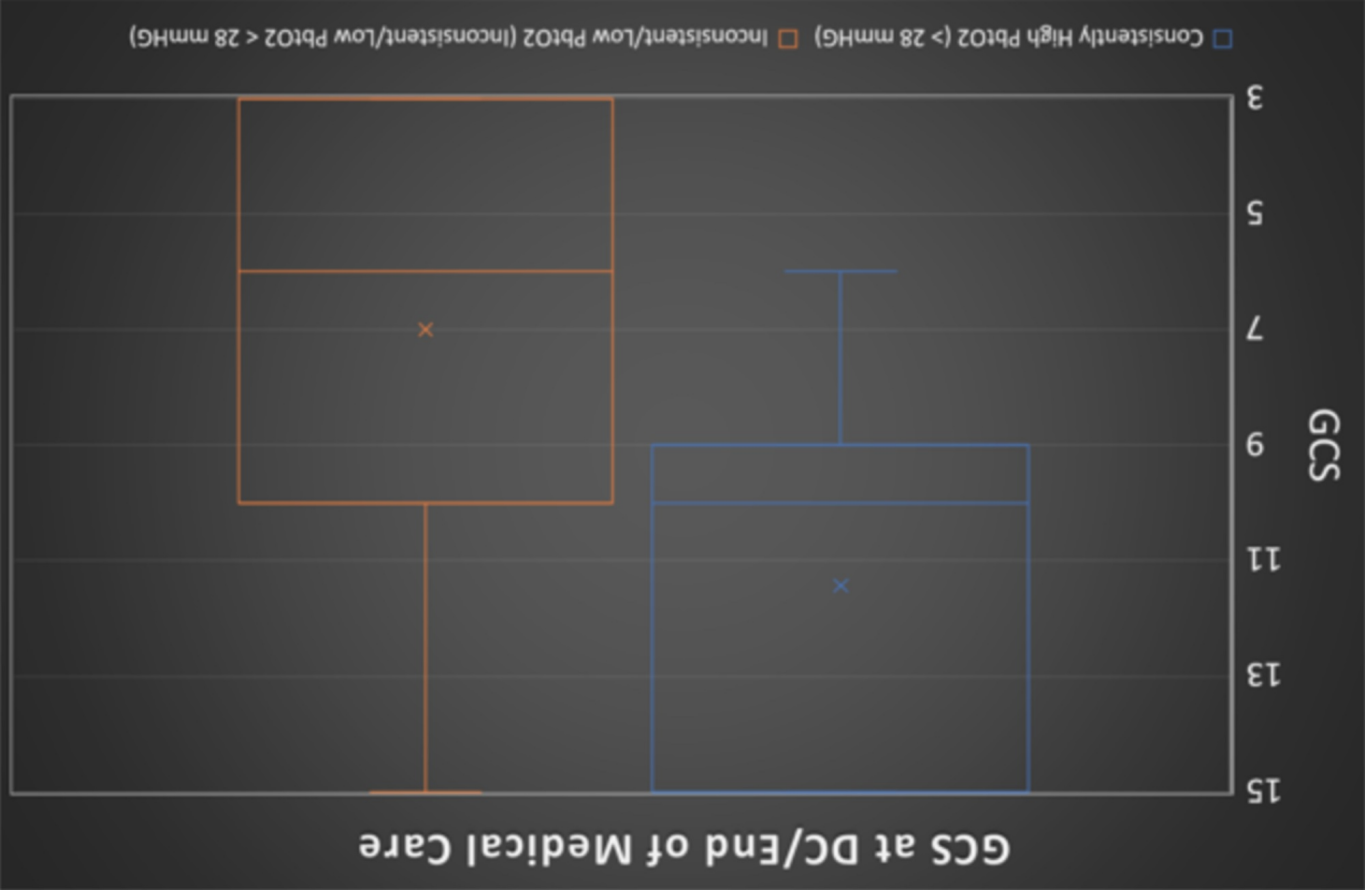
Boxplot displaying GCS for the consistently high PbtO2 group (blue) and the inconsistent/low PbtO2 group (orange) Average GCS for the consistently high group was 11.4 (n = 7). Average GCS for the inconsistent/low group was 7.0 (n=8). Glasgow Coma Scale (GCS), brain tissue oxygenation (PBtO2)

Those with consistent Days 2-5 PbtO2 scores of ≥ 28 mmHg had significantly higher GCS scores at discharge or end of ICU-level care. In fact, those in the consistently high averaged PbtO2 group had a mean GCS of 11 as compared to a GCS of 7 (Figure 3). This was found to be a statistically significant difference with GCS for the consistently high PbtO2 group being 63% higher than found in the inconsistently high/low group (p = 0.03). 

The average maximum and minimum PbtO2 for the group displaying worse outcomes was 23.0 mmHg and 14.0 mmHg, respectively. The group of patients displaying better outcomes in regard to GOS and GCS had an average maximum and minimum PbtO2 of 37.5 and 25.6 mmHg, respectively. GOS was significantly higher in those with consistently higher PbtO2 (≥ 28 mmHg) than those with lower PbtO2 scores (< 28 mmHg). The averages were 3.5 in the consistently high PbtO2 group compared to 2 in the inconsistently high/low PbtO2 group. The GOS for the >28 group was 62% higher than found in the <28 group (p = 0.01).

External ventricular drain (EVD) length actually increases for those with consistently higher PbtO2. EVD length average is 20 days in the consistently high PbtO2 group as compared to 12 days in the inconsistent/low PbtO2 group. Those patients with consistently ≥ 28 mmHg PbtO2 scores had significantly longer hospital stays (average for ≥ 28 mmHg was 40 days compared with 12 days for those in the inconsistent/low PbtO2 group). Lastly, the consistently higher PbtO2 group had fewer deaths. Out of the 15 patients, three died, of which two had consistently low PbtO2 scores based on our threshold of 28 mmHg.

Our threshold of 28 mmHg was also compared to 25 mmHg of PbtO2. We found that the length of EVD was not significantly longer (average 17.8 versus 13.5 days; p = 0.23). GOS is significantly higher in the higher PbtO2 (≥ 28 mmHg) group (average 3.4 versus 2; p < 0.001). ICU stay (days) was not significantly different (39.8 versus 24.5; p = 0.14). GCS was also found to be significantly higher in the 28 mmHg PbtO2 group (average 11.3 versus 6.5; p = 0.03). Lastly, we compared our threshold of 28 mmHg to 20 mmHg of PbtO2. We found that GOS was significantly higher in the higher PbtO2 (≥ 28 mmHg) group (average 3.3 versus 1.8; p = 0.01). GCS on discharge was significantly higher in the good group (average 11 versus 6.1; p = 0.04). ICU stay (days) was not significantly different (average 39 versus 23; p = 0.12). The length of time for EVD placement was significantly higher in the good group (average 19.3 versus 9.8; p = 0.02).

## Discussion

Brain tissue oxygenation has become an important tool in the ICU to direct TBI patient interventions and to optimize treatment options. The management of TBI patients involves continued assessment and monitoring to prevent secondary injuries, such as hypotension and hypoxia after the initial irreversible primary brain injury has occurred. Prompt assessment of cerebral oxygenation is important because studies have shown that patients are most at risk for cerebral ischemia during the first few days following injury [5].

At our institution, we consider a partial oxygen pressure above 20 mmHg to be indicative of a functional outcome and aim for readings above 28 mmHg for a good outcome [9]. In this study, we collected maximum and minimum PbtO2 data over the first five days following insertion. This was based on the recommendations provided by Integra LifeSciences technical literature directions for the use of the complete brain probe kit, which recommended a maximum duration of monitoring of five days [10]. Some authors recommend a calibration period for cerebral oxygen monitoring probes of up to 48 hours [11]. Others have found a drift in readings occurring approximately seven days after monitoring begins [12]. Prior studies attempting to define a threshold for PbtO2 have led to the conclusion that values of 19-23 mmHg should be considered associated with cerebral ischemia [13].

In our study, patients were separated by those with consistently high post Day 2 PbtO2 (> 28 mmHg) measurements from those with inconsistent and/or lower post Day 2 PbtO2 scores (< 28 mmHg) (Table 1). We chose PbtO2 values beginning at Day 2 in order to allow the Licox brain tissue monitoring device a period of calibration. We set the minimum requirement to be binned into the consistently high PbtO2 group as having at least > 28 mmHg PbtO2 maximum for Days 3, 4, and 5. This is the defined threshold below which to treat at our institution. There were seven patients in the consistently high PbtO2 category (>28 mmHg), and eight patients in the inconsistent/low PbtO2 category (<28 mmHg). After performing a t-test, we noted that the average maximum and minimum PbtO2 for the group displaying worse outcomes was 23.0 mmHg and 14 mmHg, respectively. The group of patients displaying better outcomes in regard to GOS and GCS had a maximum and minimum PbtO2 of 37.5 mmHg and 25.6 mmHg, respectively.

GOS was significantly higher in those with consistently higher PbtO2 (> 28 mmHg) than those with lower PbtO2 scores (< 28 mmHg). The average was 3.5 in the consistently high PbtO2 group as compared to 2 in the inconsistently high/low PbtO2 group. GOS for the >28 group was 62% higher than found in the < 28 group (p = 0.01).

Controversy still exists as to the best placement of brain tissue oxygen monitor probes. Uninjured brain, injured brain, and penumbra surrounding the injured brain have all been advocated for, in regard to the placement of the probe. Some authors have waited two hours to record the first PbtO2 levels to allow the calibration of probes. Placement in all 15 patients was within the white matter of the frontal lobe in an uninjured brain. The Licox system is able to measure both oxygen and temperature in the intracranial cavity, with the intention of providing additional metrics to inform clinical decision-making. Potential interventions include the augmentation of the ventilator settings, augmentation in CPP, and the addition of sedation [14]. The potential side-effects of each of these interventions must be tempered by the improvement of PbtO2 and the subsequent improvement of clinical outcomes (GCS and GOS) that may be expected to occur. Patients in our study had ventilator augmentation in the form of increasing positive end-expiratory pressure (PEEP) and/or fraction of inspired oxygen (FiO2) percent levels, performed for PbtO2 levels below 28 mmHg. Complication rates from brain tissue oxygen monitoring devices are low. However, one disadvantage of brain tissue oxygen monitoring to these patient populations includes the large artifact produced on MRI [15]. We experienced no complications in the placement in our study population.

The consistently higher PbtO2 group had fewer deaths. Out of the 15 patients, three died, of which two had consistently low PbtO2 scores, based on our threshold of 28 mmHg. This was in line with previous studies with a threshold similar to ours for PbtO2 levels. Erikkson et al. found that PbtO2 levels below 29 mmHg in the first 72 hours predicted increased mortality in TBI patients [12]. Though this study looked at mortality and included data points over a 72-hour period, our study looks at outcomes in the form of GCS and GOS and includes five days of PbtO2 data. To our knowledge, this is the first study to assess GCS and GOS in patients kept at or above 28 mmHg PbtO2 in the first five days.

We did find that EVD length and hospital stay actually increases for those with consistently higher PbtO2. EVD length average was 20 days in the consistently high PbtO2 group compared to 12 days in the inconsistent/low PbtO2 group. Those patients with consistently high PbtO2 scores had significantly longer stays with an average of 40 days as compared with 12 days for those in the inconsistent/low PbtO2 group. We hypothesize that this increased length is a result of both increased mortality in the inconsistent or low PbtO2 groups, as well as more aggressive care in those patients who were deemed to have a better prognosis.

The limitations of this study include the limited data points for PbtO2 as compared to similar studies in which PbtO2 is recorded by the minute. Additionally, our study would benefit from an increased patient population. Future studies may take into account these features to further delineate thresholds for PbtO2 treatment.

## Conclusions

Along with ICP monitors and monitoring in the assessment of CPP, brain tissue oxygenation allows yet another metric by which to optimize treatment in TBI patients. Previous methods of assessment of cerebral hypoxia relied on jugular bulb oximetry. Here, we describe the protocol used at our institution after the placement of brain tissue oxygen monitoring probes. We sought to keep PbtO2 levels above 28 mmHg for a good functional outcome. We demonstrate improvements in both GCS and GOS for patients who consistently had maximum PbtO2 levels above 28 mmHg on Days 3 to 5 while the monitoring system was in place. In addition, patients in this group had fewer deaths, in line with previous studies. However, this population did have a longer EVD duration as well as hospital stay duration. Future studies to better delineate thresholds should be performed.

## Figures and Tables

**Figure 4:**
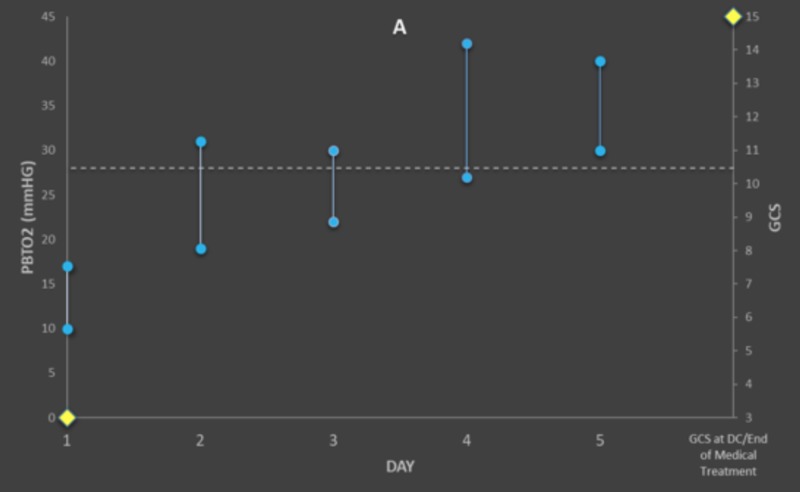
Graph for Patient A, demonstrating maximum and minimum PbtO2 levels above the pre-defined 28 mmHg threshold (dotted line) GCS during the first day of monitoring and at the hospital discharge or the end of ICU-level care are demonstrated by yellow diamonds. brain tissue oxygenation (PbtO2); Glasgow Coma Scale (GCS); intensive care unit (ICU)

**Figure 5:**
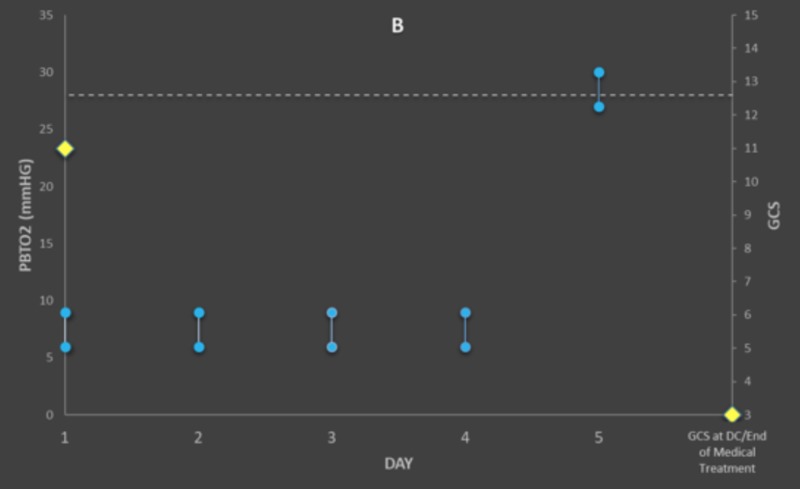
Graph for Patient B, demonstrating maximum and minimum PbtO2 levels above the pre-defined 28 mmHg threshold (dotted line) GCS during the first day of monitoring and at the hospital discharge or the end of ICU-level care are demonstrated by yellow diamonds. brain tissue oxygenation (PbtO2); Glasgow Coma Scale (GCS); intensive care unit (ICU)

**Figure 6:**
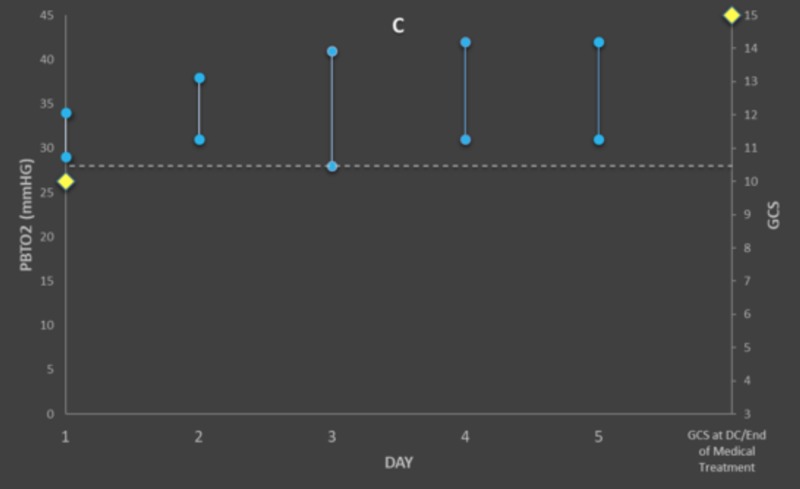
Graph for Patient C, demonstrating maximum and minimum PbtO2 levels above the pre-defined 28 mmHg threshold (dotted line) GCS during the first day of monitoring and at the hospital discharge or the end of ICU-level care are demonstrated by yellow diamonds. brain tissue oxygenation (PbtO2); Glasgow Coma Scale (GCS); intensive care unit (ICU)

**Figure 7:**
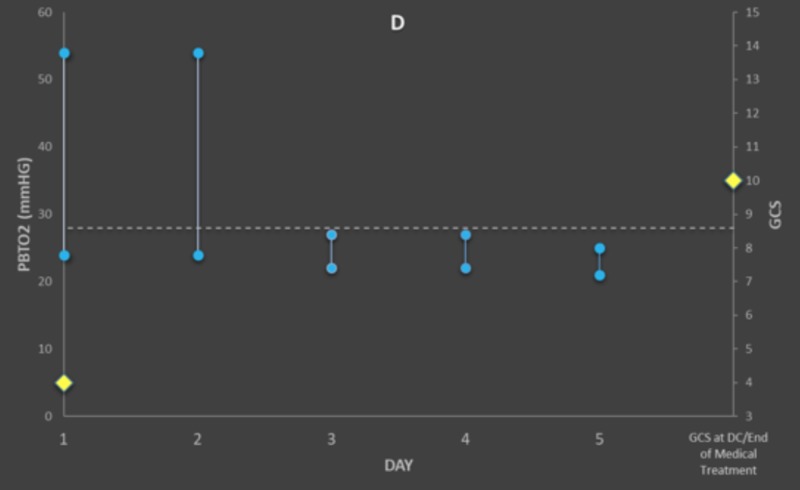
Graph for Patient D, demonstrating maximum and minimum PbtO2 levels above the pre-defined 28 mmHg threshold (dotted line) GCS during the first day of monitoring and at the hospital discharge or the end of ICU-level care are demonstrated by yellow diamonds. brain tissue oxygenation (PbtO2); Glasgow Coma Scale (GCS); intensive care unit (ICU)

**Figure 8:**
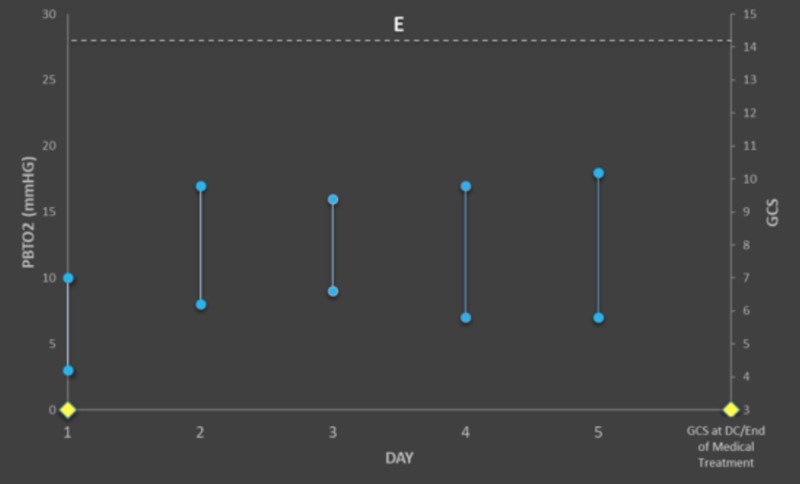
Graph for Patient E, demonstrating maximum and minimum PbtO2 levels above the pre-defined 28 mmHg threshold (dotted line) GCS during the first day of monitoring and at the hospital discharge or the end of ICU-level care are demonstrated by yellow diamonds. brain tissue oxygenation (PbtO2); Glasgow Coma Scale (GCS); intensive care unit (ICU)

**Figure 9:**
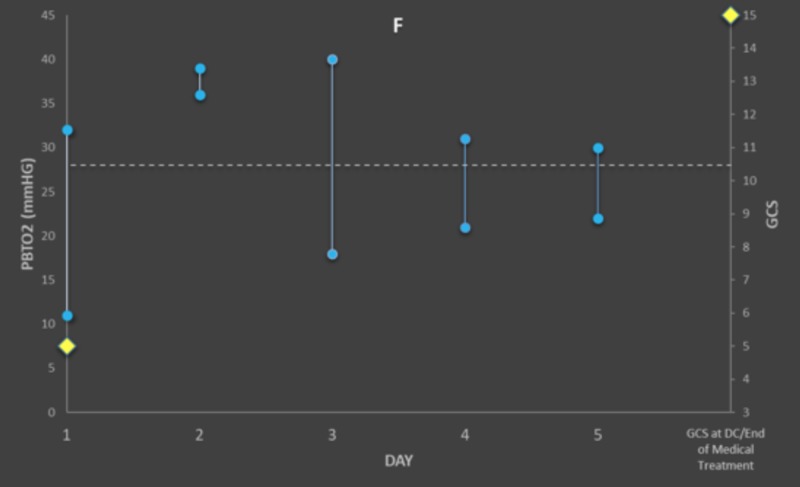
Graph for Patient F, demonstrating maximum and minimum PbtO2 levels above the pre-defined 28 mmHg threshold (dotted line) GCS during the first day of monitoring and at the hospital discharge or the end of ICU-level care are demonstrated by yellow diamonds. brain tissue oxygenation (PbtO2); Glasgow Coma Scale (GCS); intensive care unit (ICU)

**Figure 10:**
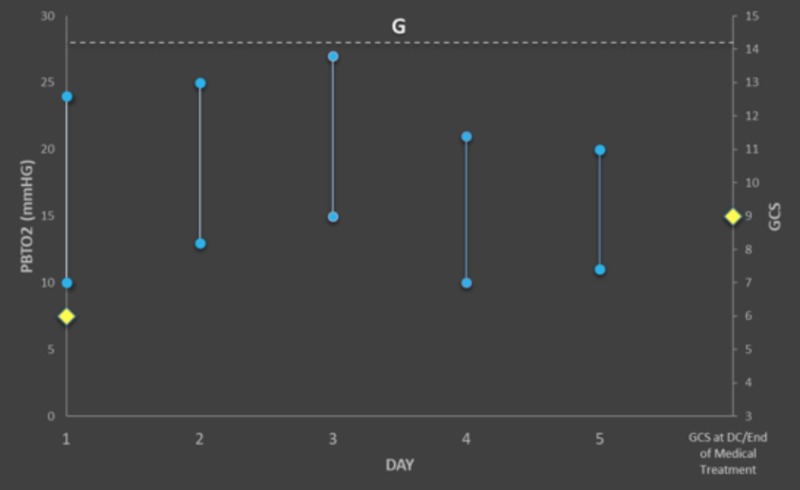
Graph for Patient G, demonstrating maximum and minimum PbtO2 levels above the pre-defined 28 mmHg threshold (dotted line) GCS during the first day of monitoring and at the hospital discharge or the end of ICU-level care are demonstrated by yellow diamonds. brain tissue oxygenation (PbtO2); Glasgow Coma Scale (GCS); intensive care unit (ICU)

**Figure 11:**
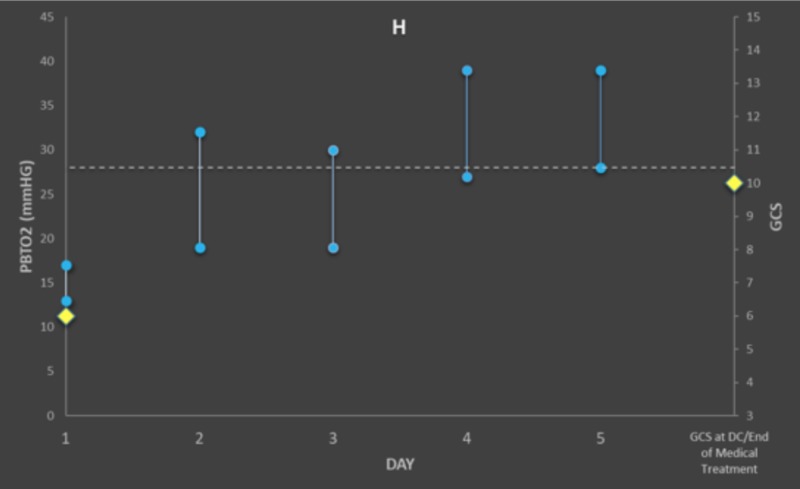
Graph for Patient H, demonstrating maximum and minimum PbtO2 levels above the pre-defined 28 mmHg threshold (dotted line) GCS during the first day of monitoring and at the hospital discharge or the end of ICU-level care are demonstrated by yellow diamonds. brain tissue oxygenation (PbtO2); Glasgow Coma Scale (GCS); intensive care unit (ICU)

**Figure 12:**
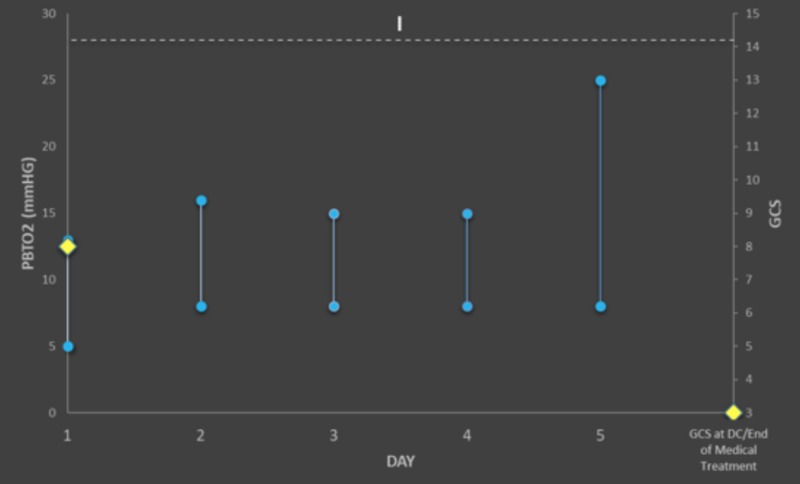
Graph for Patient I, demonstrating maximum and minimum PbtO2 levels above the pre-defined 28 mmHg threshold (dotted line) GCS during the first day of monitoring and at the hospital discharge or the end of ICU-level care are demonstrated by yellow diamonds. brain tissue oxygenation (PbtO2); Glasgow Coma Scale (GCS); intensive care unit (ICU)

**Figure 13:**
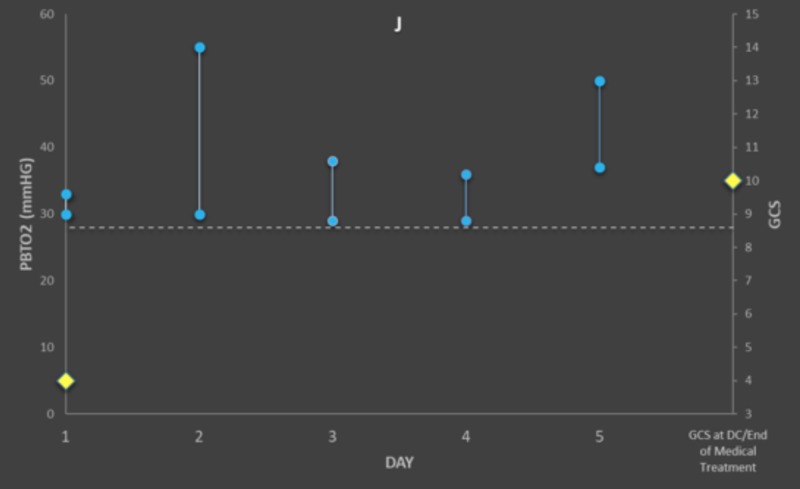
Graph for Patient J, demonstrating maximum and minimum PbtO2 levels above the pre-defined 28 mmHg threshold (dotted line) GCS during the first day of monitoring and at the hospital discharge or the end of ICU-level care are demonstrated by yellow diamonds. brain tissue oxygenation (PbtO2); Glasgow Coma Scale (GCS); intensive care unit (ICU)

**Figure 14:**
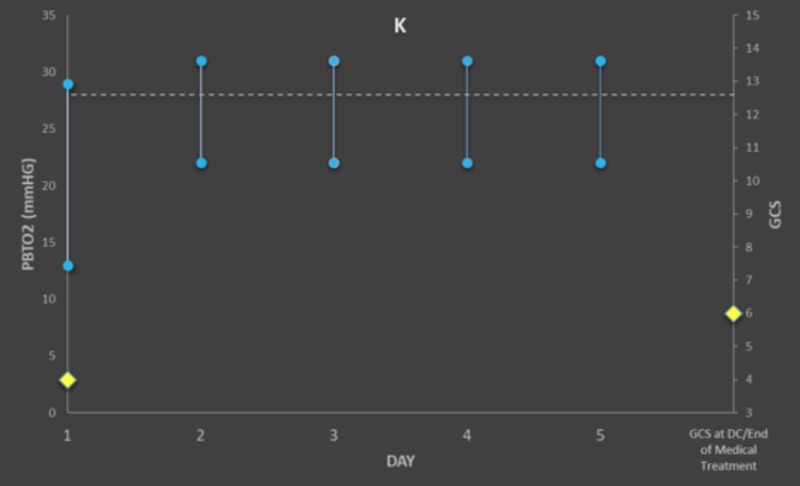
Graph for Patient K, demonstrating maximum and minimum PbtO2 levels above the pre-defined 28 mmHg threshold (dotted line) GCS during the first day of monitoring and at the hospital discharge or the end of ICU-level care are demonstrated by yellow diamonds. brain tissue oxygenation (PbtO2); Glasgow Coma Scale (GCS); intensive care unit (ICU)

**Figure 15:**
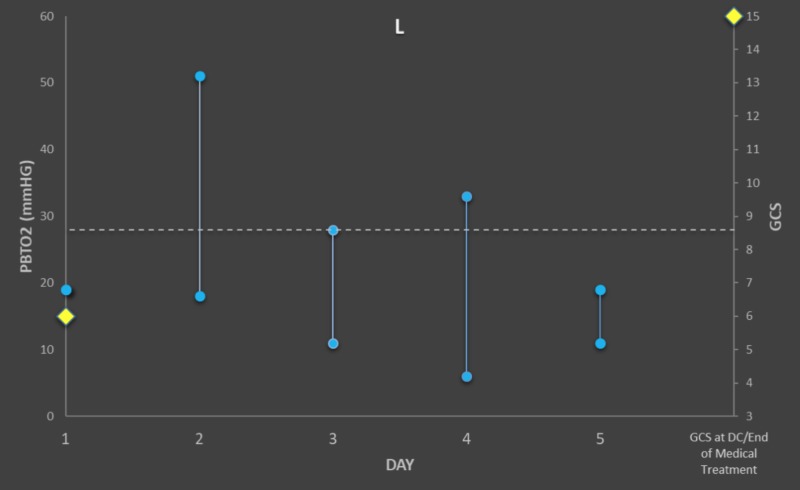
Graph for Patient L, demonstrating maximum and minimum PbtO2 levels above the pre-defined 28 mmHg threshold (dotted line) GCS during the first day of monitoring and at the hospital discharge or the end of ICU-level care are demonstrated by yellow diamonds. brain tissue oxygenation (PbtO2); Glasgow Coma Scale (GCS); intensive care unit (ICU)

**Figure 16:**
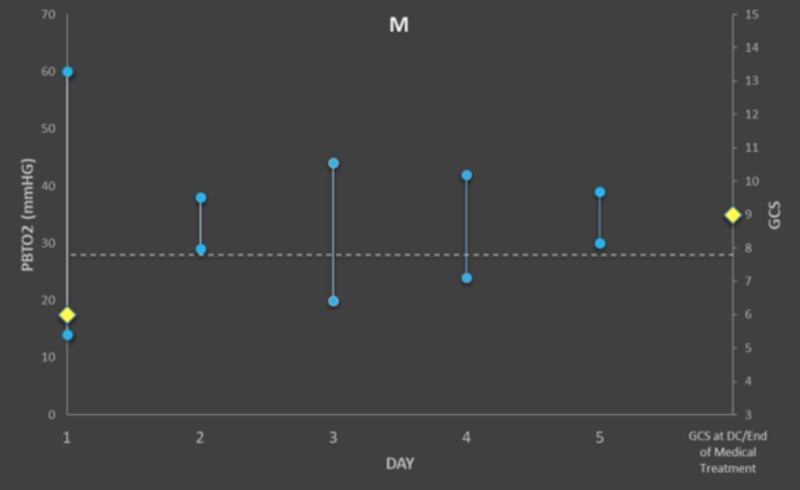
Graph for Patient M, demonstrating maximum and minimum PbtO2 levels above the pre-defined 28 mmHg threshold (dotted line) GCS during the first day of monitoring and at the hospital discharge or the end of ICU-level care are demonstrated by yellow diamonds. brain tissue oxygenation (PbtO2); Glasgow Coma Scale (GCS); intensive care unit (ICU)

**Figure 17:**
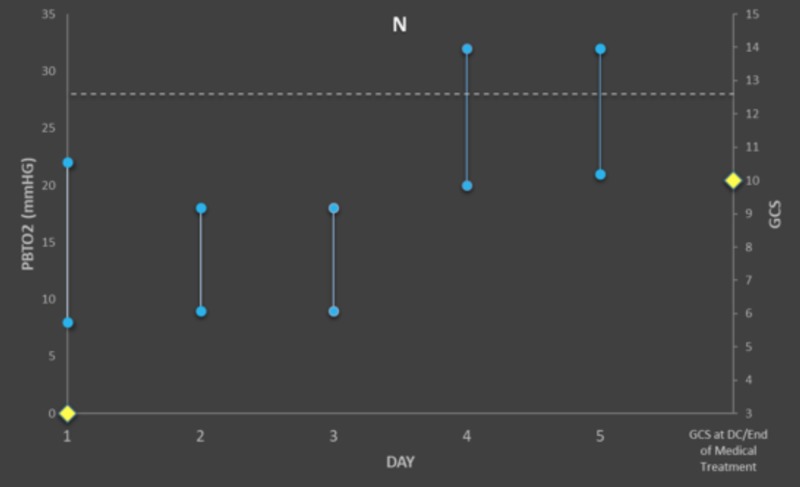
Graph for Patient N, demonstrating maximum and minimum PbtO2 levels above the pre-defined 28 mmHg threshold (dotted line) GCS during the first day of monitoring and at the hospital discharge or the end of ICU-level care are demonstrated by yellow diamonds. brain tissue oxygenation (PbtO2); Glasgow Coma Scale (GCS); intensive care unit (ICU)

**Figure 18:**
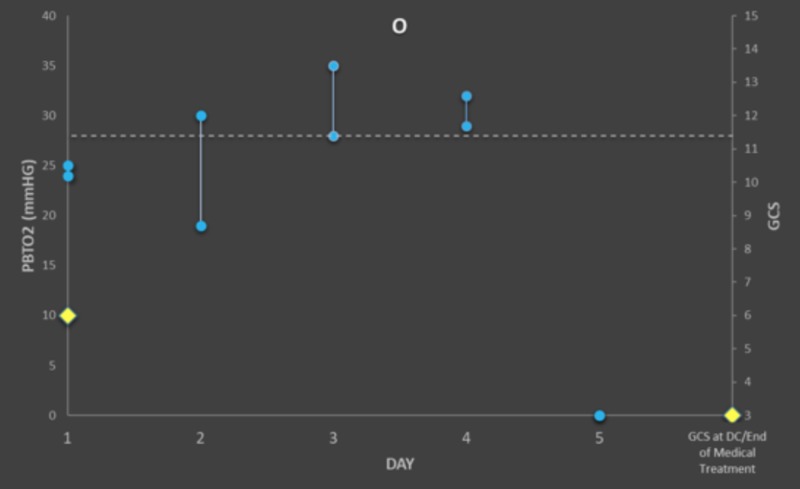
Graph for Patient O, demonstrating maximum and minimum PbtO2 levels above the pre-defined 28mmHg threshold (dotted line) GCS during the first day of monitoring and at the hospital discharge or the end of ICU-level care are demonstrated by yellow diamonds. brain tissue oxygenation (PbtO2); Glasgow Coma Scale (GCS); intensive care unit (ICU)
